# Activation of the gut microbiota-kynurenine-liver axis contributes to the development of nonalcoholic hepatic steatosis in nondiabetic adults

**DOI:** 10.18632/aging.203460

**Published:** 2021-09-02

**Authors:** Guoyuan Sui, Lianqun Jia, Dongmei Quan, Na Zhao, Guanlin Yang

**Affiliations:** 1 Key Laboratory of Ministry of Education for Traditional Chinese Medicine Viscera-State Theory and Applications, Liaoning University of Traditional Chinese Medicine, Shenyang, Liaoning, People’s Republic of China; 2 The Sixth People’s Hospital of Shenyang, Shenyang, Liaoning, People’s Republic of China

**Keywords:** gut microbiota, *Collinsella*, kynurenine, nonalcoholic hepatic steatosis, nondiabetic adults

## Abstract

The contribution of gut-liver signaling to the development of non-alcoholic hepatic steatosis (NHS) in non-diabetic adults remains unclear. We therefore performed comprehensive 16S ribosomal RNA sequencing and fecal metabolomics analyses in 32 controls and 59 non-diabetic adults with NHS and performed fecal microbiota transplantation into germ-free mice using controls and NHS patients as donors. Compared to controls, the abundance of the genera *Collinsella* and *Acinetobacter* were higher, while that of *Lachnospira* was lower, in NHS subjects. Fecal metabolomics analysis showed decreased L-tryptophan levels and increased abundance of the tryptophan metabolite kynurenine in individuals with NHS. Correlation analysis showed that kynurenine levels positively associated with the abundance of *Collinsella* and *Acinetobacter*. ROC analysis demonstrated that the combination of tryptophan and kynurenine could discriminate NHS patients from controls with good statistical power [*P* < 0.05; AUC = 0.833 (95% CI, 0.747 to 0.918)]. Supporting a key role of dysbiotic gut microbiota in NHS development, incipient hepatic steatosis and increased kynurenine levels were observed in GF mice colonized with samples from NHS patients. These results indicate that enhanced kynurenine production resulting from altered gut microbiota composition contributes to NHS in nondiabetic adults and suggest the relevance of tryptophan metabolites as diagnostic biomarkers.

## INTRODUCTION

Nonalcoholic hepatic steatosis (NHS) is the most common liver disease in the world and is associated with increased the risk of cardiovascular disease, type-2 diabetes, and liver-related complications such as hepatocellular carcinoma [1–3]. The occurrence and progression of NHS result from a combination of multiple genetic and environmental factors [1]. However, the factors that are currently known represent only a small fraction of those involved in disease onset and progression. Thus, the mechanisms underlying the pathogenesis of NHS are not fully understood, which limits the development of drugs for this disease.

Several studies have explored the characteristics of the gut microbiota in patients with NHS [1, 4]. Compared to healthy individuals, a consistently altered microbiome signature, characterized by increased abundance of *Proteobacteria*, *Enterobacteriaceae*, *Dorea*, and *Escherichia* and decreased abundance of *Ruminococcaceae*, *Rikenellaceae*, and *Coprococcus*, is found in NHS patients [4–9]. However, large discrepancies are still observed across studies [4, 5, 7–10], which may be attributed to the heterogeneity arising from distinct geographical regions, ethnicity, and population characteristics [4]. Long-term hyperglycemia or medications such as metformin were in turn reported to be important confounding factors [5].

Accumulating evidence indicates that the gut microbiota affects the health of the host by activating signaling along the gut-liver, gut-brain, gut-renal, and gut-lung axes [11–14]. Signaling through the gut-liver axis plays important roles in NHS by regulating glucose, lipid, and amino acid metabolism [5, 15–17]. In this regard, several metabolites produced by gut bacteria, notably short-chain fatty acids (SCFAs), as well as metabolic intermediates of the tryptophan and phenylalanine degradation cycles, have been associated with hepatic steatosis risk and development [5, 15–17]. However, the mechanisms underlying gut-liver signaling pathways relevant to NHS in nondiabetic adults are still far from clear. Through an integrative multi-omics approach combining clinical phenotyping, gut microbiota analysis, fecal metabolomics, and fecal microbiota transplantation (FMT) assays in germ-free (GF) mice, the present study highlights the potential contribution of gut microbiota changes and differentially expressed microbial-associated metabolites to NHS pathology in nondiabetic adults.

## RESULTS

### Characteristics of the study participants

Sex distribution, but not age, was significantly different between controls and NHS patients (*P* < 0.05). Body mass index (BMI), triglyceride (TG), total cholesterol (TC), high-density lipoprotein cholesterol (HDL-C), low-density lipoprotein cholesterol (LDL-C), fasting blood glucose, alanine aminotransferase (ALT), aspartate aminotransferase (AST), gamma glutamyl transferase (GGT), and uric acid were higher in NHS patients than in controls (*P* < 0.05) (Supplementary Table 1).

### Gut microbiota composition is altered in NHS patients

**Figure 1:**
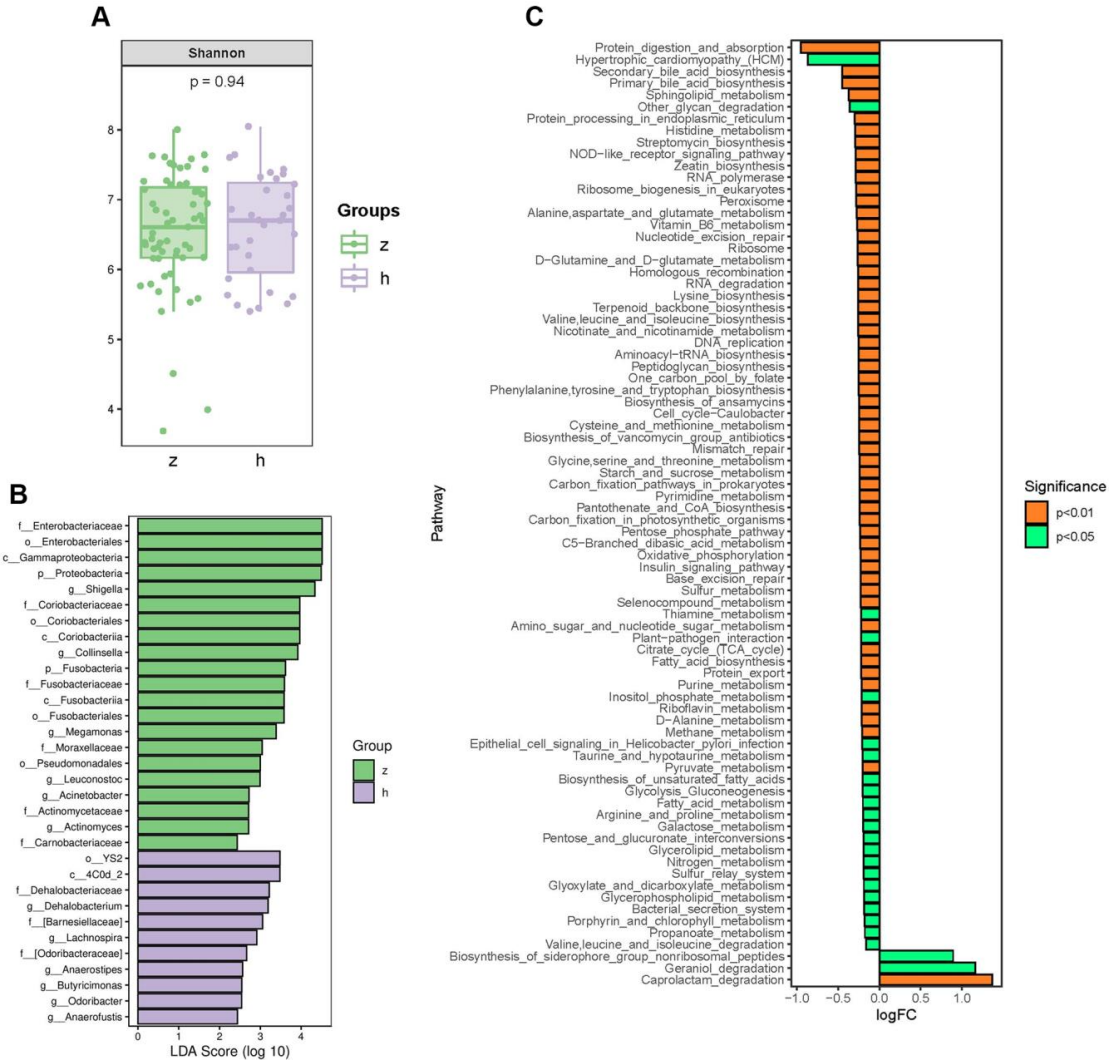
**Analysis of bacterial community structure by 16S rRNA sequencing.** (**A**) Alpha diversity analysis of gut microbiome in control and NHS stool samples. (**B**) LefSe analysis of gut microbiota composition in NHS. (**C**) KEGG pathway analysis of differentially abundant gut microbiota between control and NHS; h: control group; z: NHS group.

To identify whether NHS is associated with changes in gut microbiota composition, we performed amplification of variable regions 3 and 4 of the 16S rRNA gene in fecal samples from 91 Chinese individuals. Species diversity, indicated by the Shannon index, showed no significant differences between groups (Figure 1A). However, marked changes in the gut microbiota composition of NHS patients were revealed by taxon-based analysis. Linear discriminant analysis effect size (LefSe) showed that the abundance of phyla *Proteobacteria* and *Fusobacteria* was higher in NHS than in controls. The abundance of families *Enterobacteriaceae*, *Coriobacteriaceae*, *Fusobacteriaceae*, *Moraxellaceae*, *Actinomycetaceae*, and *Carnobacteriaceae* was higher, while the abundance of the *Dehalobacteriaceae* family was lower, in NHS compared to control samples. Genera analysis showed in turn increased abundance of *Shigella*, *Collinsella*, *Megamonas*, *Leuconostoc*, *Acinetobacter*, and *Actinomyces* and decreased abundance of *Lachnospira*, *Anaerostipes*, *Butyricimonas*, *Odoribacter*, *Anaerofustis*, and *Dehalobacterium* in NHS subjects relative to controls (Figure 1B).

Logistic regression analyses with steatosis as the dependent variable showed that the changes observed in the family *Coriobacteriaceae* and the genera *Collinsella*, *Acinetobacter*, and *Lachnospira* were still significant after adjusting for age, sex, and BMI (*P* < 0.05) (Supplementary Table 2). Based on self-reported dietary intake data, *Collinsella* abundance was associated with intake of processed meat, meat, and beverages, while *Acinetobacter* abundance was associated with intake of meat (*P* < 0.05) (Supplementary Figure 1).

### Functional alterations in gut microbiota from NHS patients

To identify functional NHS-related changes in gut microbiota, the Welch’s *t*-test was applied to examine group differences in the relative abundance of Kyoto Encyclopedia of Genes and Genomes (KEGG) pathways. Significantly differentially enriched pathways between controls and NHS subjects included protein digestion and absorption; alanine, aspartate and glutamate metabolism; histidine metabolism; valine, leucine and isoleucine biosynthesis; valine, leucine and isoleucine degradation; glycine, serine and threonine metabolism; lysine biosynthesis; cysteine and methionine metabolism; D-alanine metabolism; arginine and proline metabolism; phenylalanine, tyrosine and tryptophan biosynthesis; secondary bile acid biosynthesis; and primary bile acid biosynthesis (*P* < 0.05) (Figure 1C).

### Association of gut microbiota with clinical indices

**Figure 2:**
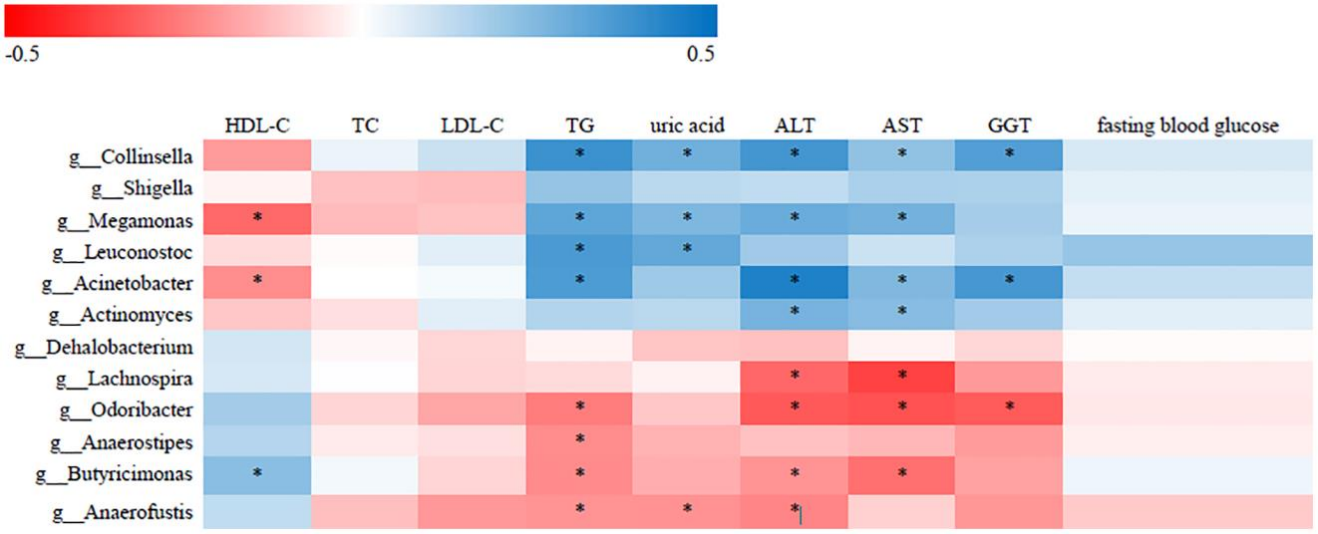
**Correlative relationships between discriminatory gut microbiota and clinical indices.** X-axis: clinical indices; Y-axis: genus; color scale represents Spearman’s correlation coefficient; red denotes strong negative correlations; blue denotes strong positive correlations; ^*^P < 0.05).

To identify correlations between gut microbiota patterns and clinical indices, we performed Spearman’s rank correlation analysis. As shown in Figure 2, the genus *Collinsella* had positive associations with TG, uric acid, ALT, AST, and GGT; the genus *Acinetobacter* was positively associated with TG, HDL-C, ALT, AST, and GGT; and the *Lachnospira* genus was negatively associated with ALT and AST (*P* < 0.05).

### Fecal metabolomics analysis

**Figure 3:**
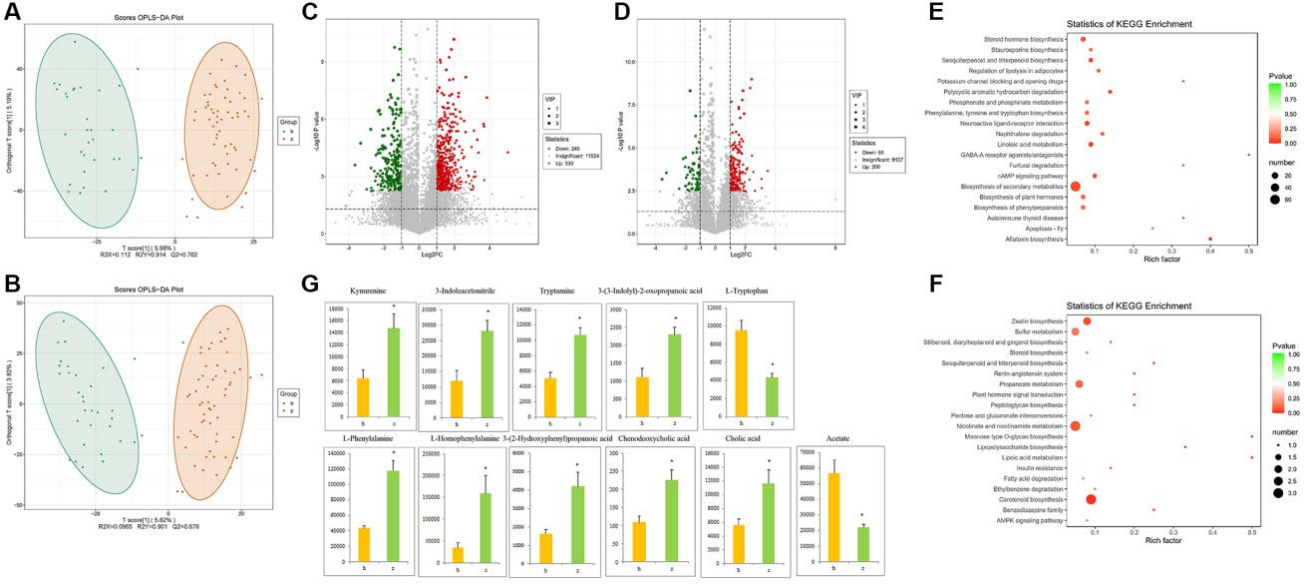
**Fecal metabolomics analysis.** (**A**) OPLS-DA score plots in positive ion mode. (**B**) OPLS-DA score plots in negative ion mode. (**C**) Differentially regulated metabolites in positive ion mode. (**D**) Differentially regulated metabolites in negative ion mode. (**E**–**F**) KEGG pathway analysis of differentially expressed metabolites in positive and negative ion modes. (**G**) Representative differential metabolites. Data are mean ± SE; h: control group; z: NHS group; ^*^P < 0.05.

To investigate more comprehensively the microbe-host interactions with potential impact on NHS, we conducted fecal metabolic profiling in the 91 study subjects by LC-MS/MS, using both positive and negative ion modes. Supervised clustering based on orthogonal partial least square-discriminant analysis (OPLS-DA) was then performed to discriminate the metabolic profiles across groups. OPLS-DA score plots for fecal samples from the NHS and control groups are shown in Figure 3A and 3B. There were 530 upregulated and 240 downregulated metabolites in positive ion mode and 200 upregulated and 68 downregulated metabolites in negative ion mode between NHS and controls (Figure 3C and 3D). KEGG analysis revealed that ‘phenylalanine, tyrosine and tryptophan biosynthesis’ and ‘biosynthesis of secondary metabolites’ were the main pathways enriched by the differentially expressed metabolites (Figure 3E and 3F). Based on the results of our KEGG pathway analysis of gut microbiota and fecal metabolomics, as well as published reports, we focused on phenylalanine, tyrosine, and tryptophan biosynthesis and metabolism, bile acid biosynthesis and metabolism, and SCFAs. With this criterion, representative differentially upregulated metabolites included kynurenine, 3-indoleacetonitrile, tryptamine, 3-(3-indolyl)-2-oxopropanoic acid, L-phenylalanine, L-homophenylalanine, 3-(2-Hydroxyphenyl)propanoic acid, chenodeoxycholic acid, and cholic acid, whereas representative differentially downregulated metabolites included L-tryptophan and acetate (Figure 3G).

### Associations of gut microbiota with fecal metabolites

The relationship between the 12 most differentially represented genera and the 11 representative differential metabolites identified in NHS patients was then examined by correlation analysis. Results showed that abundance of the genus *Collinsella* was positively associated with the levels of kynurenine, 3-indoleacetonitrile, tryptamine, and 3-(3-indolyl)-2-oxopropanoic acid, and negatively associated with L-tryptophan level (*P* < 0.05). Abundance of the genus *Acinetobacter* was in turn positively associated with the levels of kynurenine, 3-indoleacetonitrile, and 3-(3-indolyl)-2-oxopropanoic acid (*P* < 0.05). The genera *Collinsella* and *Acinetobacter* were both associated with tryptophan metabolism. Among the tryptophan metabolites assessed, only with 3-(3-indolyl)-2-oxopropanoic acid showed an association with diet (Supplementary Figure 2). Over 95% of tryptophan is metabolized by the kynurenine pathway. Interestingly, ROC analysis demonstrated that the combination of tryptophan and kynurenine could discriminate NHS patients from controls with good statistical power [*P*  <  0.05; AUC = 0.833 (95% CI, 0.747 to 0.918)] (Supplementary Figure 3).

**Figure 4:**
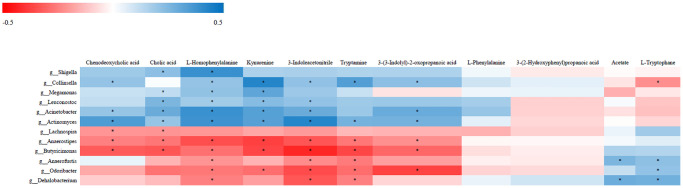
**Correlative relationships between discriminatory gut microbiota and representative fecal metabolites.** X-axis: fecal metabolites; Y-axis: genus; color scale represents Spearman’s correlation coefficient; red denotes strong negative correlations; blue denotes strong positive correlations; ^*^P < 0.05.

Chenodeoxycholic acid level was positively associated with the abundance of *Collinsella* and *Acinetobacter* and negatively associated with the abundance of *Lachnospira* (*P* < 0.05). Cholic acid level was positively associated with the abundance of *Acinetobacter* and negatively associated with the abundance of *Lachnospira* (*p* < 0.05). L-Homophenylalanine level was positively associated with the abundance of both *Collinsella* and *Acinetobacter* (*P* < 0.05) (Figure 4).

### FMT reproduces clinical and metabolic features of NHS in germ-free mice

**Figure 5:**
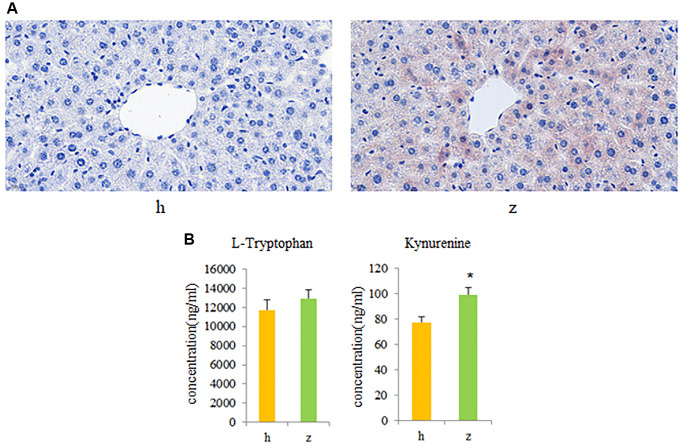
**Fecal microbiota transplantation (FMT) findings.** (**A**) Histopathological examination of liver tissue in GF mice colonized with gut microbiota from healthy controls or NHS patients (Oil Red O staining). (**B**) L-tryptophan and kynurenine quantification by UPLC-MS/MS. Data are mean ± SE; h: control group; z: NHS group; ^*^P < 0.05.

To further investigate whether activation of gut microbiota-kynurenine-liver axis contributes to the development of NHS, fecal bacteria from control and NHS patients were transplanted into GF mice. Oil Red O staining revealed significant intrahepatic lipid accumulation in GF mice colonized by gut bacteria from NHS patients, compared to mice that received gut microbiota from control subjects (Figure 5A). In addition, serum kynurenine levels were significantly increased in GF mice transplanted with NHS samples (*P* < 0.05), while no significant difference was found between the two groups regarding serum L-tryptophan levels (Figure 5B).

## DISCUSSION

Clinical and animal studies have shown that dysbiosis of the gut microbiota represents an independent risk factor for NHS [1, 5]. Indeed, since alterations the gut microbiota can contribute to insulin resistance, chronic inflammation, and disorders of glucose, lipid, and amino acid metabolism, the gut microbiota is considered an important target for NHS treatment [1, 18]. Our study showed that the phylum *Proteobacteria*, the family *Enterobacteriaceae*, and the genera *Collinsella* and *Acinetobacter* were enriched, while the abundance of the genus *Lachnospira* was decreased, in nondiabetic adults with NHS. The observed increase in the relative abundance of *Proteobacteria* and *Enterobacteriaceae* was indeed consistent with the results of previous studies [5, 8]. Research showed that *Proteobacteria* facilitate the development of hepatic steatosis by regulating endotoxin production and the immune response [19, 20]. The risk of developing NHS is also increased by overrepresentation of *Enterobacteriaceae*, which augment endogenous ethanol production [21]. Increased abundance of the genus *Collinsella* was reported in type 2 diabetes, non-alcoholic steatohepatitis, and rheumatoid arthritis, among other inflammatory conditions [22–27]. *Collinsella* (7α-dehydroxylating bacteria) was reported to metabolize bile acids to oxo-bile acid intermediates [28–30]. The production of these secondary bile acids may disrupt the intestinal mucosal barrier and participate in the development of NHS [28–30]. Accordingly, reduced *Collinsella* abundance following administration of the antibiotic rifaximin was associated with a drop in secondary fecal bile acid concentrations [30]. NHS patient samples showed also decreased abundance of *Lachnospira*, which are SCFA-producing bacteria. SCFAs have shown to confer many beneficial health effects, including maintaining the gut barrier, providing energy sources for enterocytes and colonocytes, inhibiting the proliferation of hepatic cells, and preventing inflammation [31–34].

Fecal metabolomics results showed that the levels of L-phenylalanine and cholic acid were upregulated, while those of acetate were downregulated, in fecal samples from NHS patients. L-phenylalanine, an essential amino acid, may be converted into tyrosine by phenylalanine hydroxylase [35]. Abnormal phenylalanine levels are associated with many health conditions, such as heart failure, obesity, hypertriglyceridemia, and type 2 diabetes [36–39]. Hoyles et al. found that plasma PAA, a phenylalanine-derived bacterial metabolite, was positively associated with liver steatosis severity. They further showed that mice treated with PAA for 2 weeks had significantly increased hepatic triglyceride accumulation [5]. Previous studies reported that increased cholic acid levels were associated with dyslipidemia, atherosclerosis, and non-alcoholic fatty liver disease (NAFLD) [40–44]. Cholic acid, a primary bile acid, is formed in the liver by cholesterol 7-hydroxylase (CYP7A1), the rate-limiting enzyme that regulates the synthesis of bile acids from cholesterol [44]. Yamada et al. showed that dietary cholic acid promoted NAFLD in pigs by activating local and systemic oxidative stress-induced signaling leading to macrophage mobilization [41]. Decreased production of acetate, a major intestinal and circulating SCFA produced by gut bacteria from dietary fiber metabolism, was also reported in many diseases, including NAFLD, obesity, cardiovascular disease, and type 2 diabetes [45–47]. In this regard, Sun et al. found that changes in the gut microbiota induced by a high-sucrose diet promoted the development of NAFLD in rats by reducing microbial production of SCFAs [16].

Several metabolites related to tryptophan metabolism, such as L-tryptophan, kynurenine, 3-indoleacetonitrile, tryptamine, and 3-(3-indolyl)-2-oxopropanoic acid, were also altered in fecal samples from NHS subjects. Three metabolic pathways of tryptophan in the intestine are directly and indirectly controlled by the gut microbiota: (a) direct conversion of tryptophan into several molecules, including indole-3-aldehyde (IAld) and indole-3-acetic acid (IAA); (b) the kynurenine pathway in epithelial and immune cells, initiated by indoleamine 2, 3-dioxygenase (IDO) 1; and (c) the 5-hydroxytryptamine (5-HT) production pathway in enterochromaffin cells, mediated by Trp hydroxylase 1 (TpH 1) [48]. Alterations in tryptophan metabolism are involved in the occurrence and development of many diseases, such as inflammatory bowel disease, irritable bowel syndrome, metabolic syndrome, obesity, infectious diseases, and neuropsychiatric disorders [48]. Our study revealed that fecal kynurenine level was higher in NHS patients than in controls. Correlation analysis showed that kynurenine level was positively associated with the abundance of genera *Collinsella*, *Megamonas*, *Leuconostoc*, *Acinetobacter*, and *Actinomyces* and negatively associated with abundance of the genera *Anaerostipes*, *Butyricimonas*, and *Odoribacter*. Consistently, FMT experiments showed that compared to controls, serum kynurenine was increased in GF mice colonized with gut bacteria from NHS patients. Kynurenine is a gut microbiota-derived metabolite of tryptophan via IDO1 catalysis. IDO1 upregulation is a hallmark of obesity, and its deletion or inhibition improves insulin sensitivity, preserves the gut mucosal barrier, decreases chronic inflammation, and regulates lipid metabolism in liver and adipose tissues [49]. Notably, kynurenine was shown to trigger obesity by binding to the aryl hydrocarbon receptor (AhR), and blocking IDO or AhR in mice significantly attenuated long-term high-fat diet-induced obesity and liver steatosis [50]. The abundance of the genus *Collinsella* was positively associated with the levels of kynurenine, 3-indoleacetonitrile, tryptamine, and 3-(3-indolyl)-2-oxopropanoic acid, and negatively associated with L-tryptophane. Although the association between increased *Collinsella* abundance and NAFLD has been reported [25], further work is required to determine whether *Collinsella* promotes the development of NHS in nondiabetic adults via the kynurenine pathway.

We acknowledge several limitations in this study. First, there was an unequal sex distribution in our study, which included more females in the control group and more males in the NHS group. Although our regression models were adjusted for sex, the effect of sex on gut microbiota is well established and may therefore affect our results [51]. Second, in our study sample NHS was proven by ultrasonic diagnosis. Unlike histology, which allows distinguishing between simple steatosis and steatohepatitis, ultrasonography allows for reliable and accurate detection of moderate-severe fatty liver [52].

In summary, this study revealed that alterations in the gut microbiota are associated with differential expression of fecal metabolites in nondiabetic adults with NHS. Specifically, our data supports a contributing role for kynurenine, a gut microbiota-derived tryptophan metabolite, in the pathogenesis of non-diabetic NHS and suggests that tryptophan/kynurenine levels may be reliable clinical biomarkers for this condition.

## MATERIALS AND METHODS

### Samples

A total of 91 subjects were enrolled in this case-control study between March 2019 and March 2020 at the Sixth People’s Hospital of Shenyang, China. Fifty-nine patients proven by ultrasonic diagnosis were included. The inclusion criteria were: (1) Age 18–72 years; (2) no alcohol, or alcohol consumption equivalent to <210 g and <140 g of ethanol per week, for men and women, respectively; and (3) non-alcoholic hepatic steatosis proven by ultrasonic diagnosis. The exclusion criteria were: (1) hepatic steatosis caused by alcoholic liver disease, genotype 3 hepatitis C virus infection, autoimmune hepatitis, hepatolenticular degeneration, drugs (tamoxifen, amiodarone, sodium valproate, methotrexate, glucocorticoids, etc.), total parenteral nutrition, inflammatory bowel disease, celiac disease, hypothyroidism, Cushing’s syndrome, beta lipoprotein deficiency, lipid atrophy diabetes, Mauriac syndrome, etc.; (2) long-term drinkers (≥210 g ethanol/week for men, ≥140 g ethanol/week for women); (3) liver cirrhosis, liver malignant tumor, acute biliary tract infectious disease, and those who were taking or have taken hepatotoxic Chinese medicine and/or Western medicine in the past 3 months; (4) systolic blood pressure higher than 160 mm Hg and/or diastolic blood pressure higher than 100 mm Hg after standard antihypertensive treatment; (5) malignant tumors, congenital heart disease, acute myocardial infarction, post-PCI, severe arrhythmia, acute cerebral infarction and brain bleeding, chronic obstructive pulmonary disease, pulmonary heart disease, respiratory failure, renal insufficiency (blood BUN or Cr exceeding 1.5 times the upper limit of the reference value), and hematopoietic system diseases; (6) psychiatric disorders; (7) diabetes; (8) severe diarrhea (3 or more watery stools per day in the past 3 months); (9) severe constipation (2 or fewer bowel movements per week in the past 3 months, accompanied by difficulty in defecation); (10) pregnant and lactating women; (11) subjects who have taken antibiotics, steroidal anti-inflammatory drugs and probiotics in the past 3 months; (12) diets containing probiotics such as yogurt for the past 1 week. The control group consisted of 32 age-matched healthy volunteers from the Sixth People’s Hospital of Shenyang with normal serum lipids, blood glucose, liver enzymes and abdominal ultrasonography findings. This study was approved by the Ethics Committee of the Sixth People’s Hospital of Shenyang (No.2018-05-002-02), and informed consent was obtained from all subjects.

### Biochemical measurements

Blood samples were drawn after an overnight fast from an antecubital vein. TG, TC, HDL-C, LDL-C, fasting blood glucose, ALT, AST, GGT, and uric acid were determined using biochemical kits. The experimental procedures followed the corresponding specifications.

### Diet assessment

Dietary intake assessment over the past year referred to the semi-quantitative food frequency questionnaire [53–54]. Diet items included rice, flour, dessert, fried foods, coarse grains, tubers, processed meat, meat, seafood, nuts and legumes, vegetables, mushrooms, fruits, dairy foods, beverages, and eggs. Frequency of food intake ranged from “≤1/month” to “≥3/day”. Portion size ranged from “50 g or below” to “250 g or above”. For dairy foods, portion size ranged from “50 ml or below” to “400 ml or above”; for beverages, intake per serving ranged from “100 ml” to “550 ml or above”; for eggs, portion size ranged from “half and below” to “three or above”.

### 16S ribosomal RNA (rRNA) sequencing

Total genomic DNA samples were extracted from stool samples using the OMEGA Soil DNA Kit (D5625-01) (Omega Bio-Tek, USA) according to the manufacturer’s instructions. The quality and quantity of extracted DNA were measured using agarose gel electrophoresis and a NanoDrop ND-1000 spectrophotometer (Thermo Fisher Scientific, USA), respectively. PCR amplification was performed for the V3-V4 regions of bacterial 16S rRNA genes. PCR amplicons were purified and quantified. Sequencing libraries were prepared with Illumina's TruSeq Nano DNA LT Library Prep Kit. Pair-end 2 × 250 bp sequencing was performed using the Illumina MiSeq platform. QIIME2 and R packages (v3.2.0) were used for sequence data analyses. Raw sequence data were demultiplexed using the demux plugin. Sequences were then quality filtered, denoised, and merged, and chimeras were removed using the DADA2 plugin.

### Metabolomics analysis

Stool (100 mg) was homogenized with ice-cold water (300 μl) and mixed and vortexed with cold steel balls (5 min). The homogenized stool was then added to pure methanol (500 μl), vortexed, incubated on ice (10 min), and centrifuged (12,000 rpm, 4°C, 10 min). Then, 600 μl of supernatant was added to another centrifuge tube and concentrated. The dried product was added to 5% methanol-water (100 μl), vortexed and centrifuged (12,000 rpm, 4°C, 10 min). Finally, the supernatant was used for LC-MS/MS analysis using a Waters ACQUITY UPLC HSS T3 C18 column (1.8 μm, 2.1 mm×100 mm). Spectral data were obtained in positive and negative ion modes. The original data file was converted into mzML format by Proteo Wizard software. Then, the XCMS program was used to perform peak extraction and alignment and retention time correction. Peak area was corrected by the “SVR” method and peaks with deletion rates >50% in each group of samples were filtered. Metabolic identification information was obtained by searching the public database metDNA (http://metdna.zhulab.cn) and our laboratory’s self-built database.

### GF mice and FMT procedures

Fifteen six-week-old GF male mice were fed in a sterile isolator in the Experimental Animal Center at Shanghai Shrek Experimental Animal Co., Ltd. (temperature: 20–22°C; humidity: 50–60%; day/night cycle: 12 h/12 h). Drinking bottles and cages were sterilized under high temperature and pressure (121°C, 60 min) and water, feed, and bedding materials were irradiated (50 kGy) prior to use or administration. Donors with nonalcoholic hepatic steatosis (*n* = 4, two men and two women) and control subjects (*n* = 2, one man and one woman) were randomly selected. For each group, 100 mg of combined stool samples were resuspended with a vortex in 600 μl of reduced PBS (PBS with 0.5 g/l cysteine and 0.2 g/l Na_2_S). The mixture was centrifuged (2500 rpm, 1 min) to remove insoluble materials and the supernatant was transferred to a new sterile test tube. GF mice were fed with a balanced diet for 4 weeks and gavaged once daily with 100 μL of fecal suspension over the first 4 days of this feeding cycle. On the day of sampling, the mice were anesthetized using 1% pentobarbital (0.2 ml/mouse). Mice were then sacrificed by CO_2_ euthanasia method and liver histology and serum L-tryptophan and kynurenine quantification performed as described below.

### Oil Red O staining

Liver samples were dehydrated in 15% and 30% sucrose and then embedded with optimal cutting temperature compound (OCT) to prepare frozen sections (8–10 μm). The frozen sections were stained with Oil Red O and a Nikon E100 microscope was used to acquire images.

### L-tryptophan and kynurenine quantification by UPLC–MS/MS

Standard mix solution and 50 μL serum with 50 μL protein precipitation agent (including NVL) were mixed and centrifuged (13,200 rpm, 4 min). Eight microliters of supernatant and 42 μL of labeling buffer were then mixed and centrifuged. Then, 20 μL of derivatization solution was added, centrifuged, and the sample was derivatized (55°C, 15 min). After derivatization, the sample was cooled in a refrigerator, centrifuged at 13,500 rpm instantly, and 50 μL collected for UPLC–MS/MS analysis using a Waters MSLab 45+AA-C18 column (5 μm, 150 mm × 4.6 mm).

### Statistical analysis

ASV-level alpha diversity indices (Shannon diversity index) were calculated. LEfSe was performed to detect differentially abundant taxa across groups using the default parameters (LDA Effect Size >2 and *P* < 0.05). Logistic regression was performed to investigate the association between steatosis and gut microbiota after adjusting for age, sex, and BMI. Phylogenetic reconstruction of unobserved states (PICRUSt2) was used to predict and analyze species functions according to amplicon sequencing data. For metabolomics analysis, univariate analysis (Student’s *t*-test) and multivariate analysis (orthogonal partial least squares discriminant analysis; OPLS-DA) were applied. Correlations between gut microbiota at genera level, clinical indices, and fecal metabolites were tested with Spearman’s correlation. The latter was also used to explore correlations between gut microbiota at genera level, fecal metabolites, and diet. Receiver-operating characteristic (ROC) curves were plotted and area under the ROC curve (AUC) was used as an accuracy index for evaluating the diagnostic performance of the tryptophan-kynurenine metabolites. Student’s *t*-test was used to detect differences in serum L-tryptophan and kynurenine values across groups. QIIME2, R packages (v3.2.0), and SPSS 21.0 were used for data analyses.

### Data sharing statement

The datasets used in our study are available from the corresponding author on reasonable request.

## Supplementary Materials

Supplementary Figures

Supplementary Tables
