# Supplementary material for: Activation of the gut microbiota-kynurenine-liver axis contributes to the development of nonalcoholic hepatic steatosis in nondiabetic adults
Source: Aging (Albany NY). 2021 Sep 2;13(17):21309–24. doi: 10.18632/aging.203460 (PMC8457600; doi:10.18632/aging.203460)
Supplement: Supplementary Figures [file aging-13-203460-s001.pdf]

SUPPLEMENTARY FIGURES

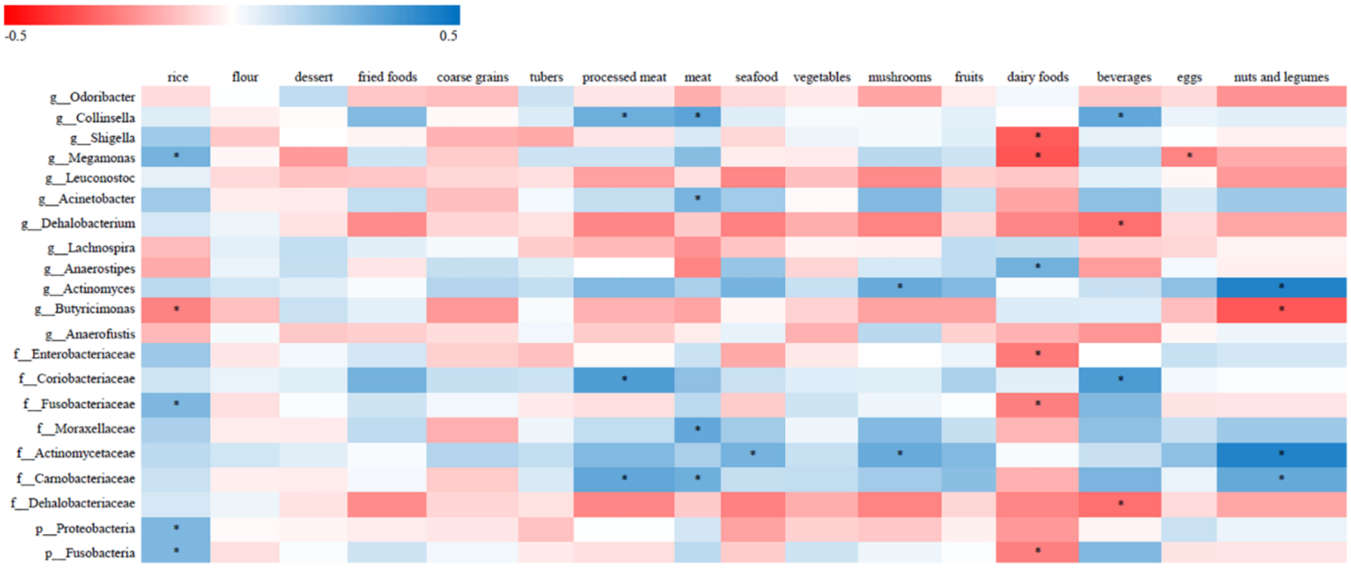

Supplementary Figure 1. Correlative relationships between discriminatory gut microbiota and diet. X-axis: diet; Y-axis: gut microbiota (phylum/family/genus); color scale represents Spearman's correlation coefficient; red denotes strong negative correlations; blue denotes strong positive correlations; $^{*}P < 0.05$.

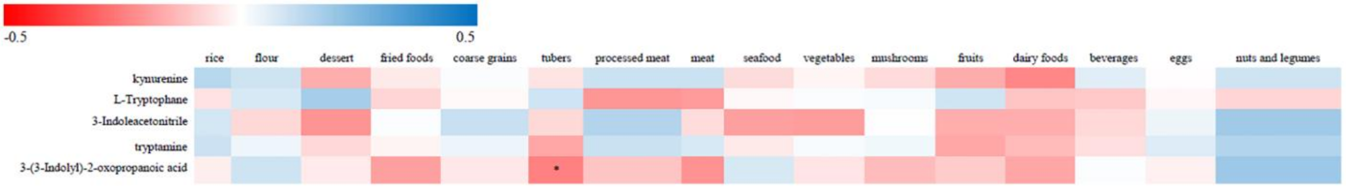

Supplementary Figure 2. Correlative relationships between discriminatory tryptophan metabolites and diet. X-axis: diet; Y-axis: tryptophan metabolites; color scale represents Spearman's correlation coefficient; red denotes strong negative correlations; blue denotes strong positive correlations; $^{*}P < 0.05$.

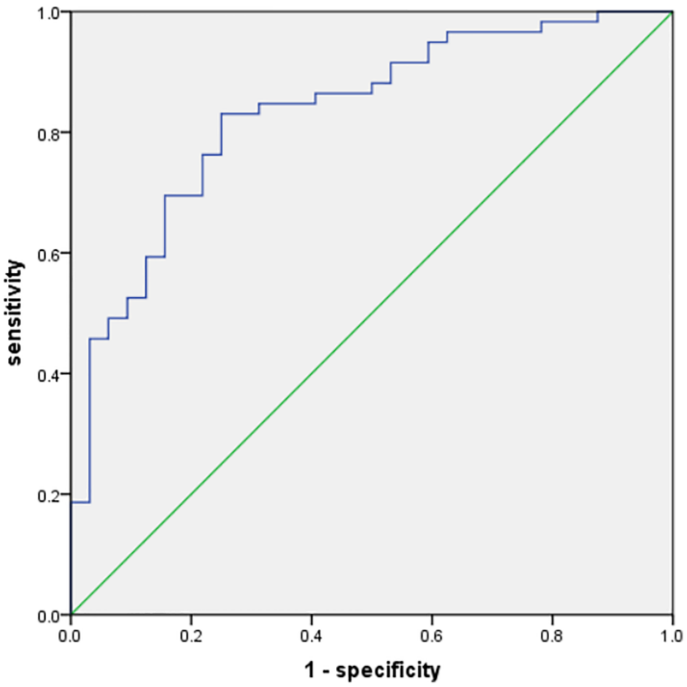

Supplementary Figure 3. ROC analysis of the combination of tryptophan and kynurenine in the diagnosis of NHS. The area under the ROC curve (AUC) is 0.833, and the 95% confidence interval (CI) is 0.747–0.918.
