# Supplementary material for: Activation of the gut microbiota-kynurenine-liver axis contributes to the development of nonalcoholic hepatic steatosis in nondiabetic adults
Source: Aging (Albany NY). 2021 Sep 2;13(17):21309–24. doi: 10.18632/aging.203460 (PMC8457600; doi:10.18632/aging.203460)
Supplement: Supplementary Tables [file aging-13-203460-s002.pdf]

## SUPPLEMENTARY TABLES

**Supplementary Table 1. Characteristics of the study participants.**

| Variables | Controls (*n* = 32) | Nonalcoholic hepatic steatosis (*n* = 59) | *P* |
|---|---|---|---|
| Sex (male/female) | (12/20) | (40/19) | $P < 0.05$ |
| age | 45.84 ± 13.83 | 49.15 ± 11.22 | $P > 0.05$ |
| BMI | 22.68 ± 2.45 | 26.62 ± 3.02 | $P < 0.05$ |
| TG | 1.00 ± 0.29 | 2.24 ± 1.32 | $P < 0.05$ |
| TC | 4.78 ± 0.60 | 5.19 ± 1.18 | $P < 0.05$ |
| HDL-C | 1.73 ± 0.45 | 1.43 ± 0.39 | $P < 0.05$ |
| LDL-C | 2.91 ± 0.60 | 3.34 ± 0.89 | $P < 0.05$ |
| fasting blood-glucose | 4.63 ± 0.43 | 4.99 ± 0.56 | $P < 0.05$ |
| ALT | 15.15 ± 9.00 | 30.49 ± 32.00 | $P < 0.05$ |
| AST | 18.17 ± 6.00 | 23.00 ± 10.00 | $P < 0.05$ |
| GGT | 18.95 ± 17.00 | 31.15 ± 39.00 | $P < 0.05$ |
| uric acid | 267.08 ± 73.08 | 350.23 ± 81.48 | $P < 0.05$ |

**Supplementary Table 2. Logistic regression analyses with steatosis as dependent variable.**

| Variables | B | SE | *P* |
|---|---|---|---|
| *Coriobacteriaceae* | 61.17 | 26.68 | $P < 0.05$ |
| *Collinsella* | 55.46 | 28.15 | $P < 0.05$ |
| *Acinetobacter* | 7690.89 | 3551.86 | $P < 0.05$ |
| *Lachnospira* | −1551.01 | 592.79 | $P < 0.05$ |

All gut microbiota (log10 (1+phylum/family/genus)) are analyzed. These models were adjusting for age, sex and BMI.
